# Targeted *Staphylococcus aureus* decolonization in acute inpatient and intensive care settings of an academic medical center

**DOI:** 10.1017/ash.2022.161

**Published:** 2022-05-16

**Authors:** David DiTullio, Courtney Takats, Sarah Hochman

## Abstract

**Background:** *Staphylococcus aureus* is a common cause of healthcare associated infections and is associated with high mortality. *S. aureus* colonization of skin and mucosa contributes to its pathogenesis. Universal *S. aureu*s decolonization reduces methicillin-resistant *S. aureus* (MRSA) and other bloodstream infections among ICU patients. However, universal decolonization in acute-care settings has not shown a similar benefit. We describe a targeted decolonization protocol implemented at a large academic hospital across acute-care and intensive care settings. We assessed the impact of decolonization on *S. aureus*–related infections. **Methods:** Adults admitted in 2018–2019 to the medicine, oncology, transplant, and ICU services were screened for *S. aureus* colonization using nasal swabs for MRSA/MSSA by culture. Those with *S. aureus* detected underwent decolonization with 5 days of chlorhexidine 2% baths and mupirocin intranasal ointment. Decolonization was considered complete if given for 5 days. The primary outcome was *S. aureus* invasive infection from hospital day 3 until discharge, defined by positive clinical cultures from sterile sites. Secondary outcomes included 30-day readmission and 30-day mortality. The control population was patients with negative MRSA/MSSA nasal screening in the same hospital units. **Results:** In total, 4,465 (23%) of 19,065 screening tests were positive for MSSA (75%) or MRSA (25%). The median age was 69 years (IQR, 56–80), and the median length of stay (LOS) was 6 days (IQR, 4–10). Among patients with LOS ≥3 days, 541 (16%) completed decolonization and 2,161 (64%) received no decolonization. The rate of complete decolonization increased to 35% among those with LOS ≥ 7 days. In total, 802 screened patients developed invasive *S. aureus* infections. Of 4,437 colonized patients, 536 (12%) had invasive infections, compared with 265 (2.1%) invasive infections in 12,917 noncolonized patients. Among patients with *S. aureus* colonization, 24% of decolonized patients developed invasive infection and 13% of patients who were not decolonized developed invasive infection. Rates of 30-day readmission and mortality were 28% and 10%, respectively, among fully decolonized patients, versus 20% and 6.6% among those receiving no decolonization. **Conclusions:** These data provide an assessment of the efficacy of a targeted screening and decolonization program. Although decolonization did not reduce rates of invasive infection or secondary outcomes, further analysis is needed. Patients with longer lengths of stay are more likely to receive full decolonization but are also at higher risk of invasive infection, which may contribute to our unexpected results.

**Funding:** None

**Disclosures:** None

